# The effect of topical administration of cyclopentolate on ocular biometry: An analysis for mouse and human models

**DOI:** 10.1038/s41598-017-09924-5

**Published:** 2017-08-30

**Authors:** Furong Huang, Shenghai Huang, Ruozhong Xie, Yanan Yang, Jiaofeng Yan, Xiaowen Cao, Chunlan Zhang, Feng Zhou, Meixiao Shen, Jia Qu, Xiangtian Zhou

**Affiliations:** 1 School of Optometry and Ophthalmology and Eye Hospital, Wenzhou Medical University, Wenzhou, Zhejiang, China; 2 State Key Laboratory of optometry, Ophthalmology, and Vision Science, P. R. China and Zhejiang Provincial Key Laboratory of Ophthalmology and Optometry, Wenzhou, Zhejiang, China

## Abstract

Mydriasis with muscarinic antagonists have been used routinely prior to retinal examination and sometimes prior to refractive measurements of the mouse eye. However, biometric changes during topical administration of muscarinic antagonists have not been fully investigated in mice and humans. We found that the mouse eyes treated with cyclopentolate developed a hyperopia with a reduction in both the vitreous chamber depth and axial length. In humans, prior to the cyclopentolate treatment, a 6D accommodative stimulus produced a myopic shift with a reduced anterior chamber depth, choroidal thickness and anterior lens radius of curvature and an increase in lens thickness. After the cyclopentolate treatment, human eyes developed a hyperopic shift with an increased anterior chamber depth and anterior lens radius of curvature and a reduced lens thickness. Therefore, the biometric changes associated with this hyperopic shift were mainly located in the posterior segment of the eye in mice. However, it is the anterior segment of the eye that plays a main role in the hyperopic shift in human subjects. These results further indicate that mouse eyes do not have accommodation which needs to be taken into account when they are used for the study of human refractive errors.

## Introduction

Mice have been used for the study of mechanisms involved in human refractive errors for at least 10 years^1, 2^. The refractive status of human eyes varies with the distance of visual targets due to existence of the accommodation. Accommodation in human eyes is a dynamic process in which the ocular system compensates for retinal defocus of near vision^3–5^. This process involves contraction of the ciliary muscle and a reduction in the tension of the zonules which attach the lens to the ciliary body, resulting in a steeper lens contour, a shallower anterior chamber depth and an increased lens thickness^6–11^. All of these changes in parts of the anterior segments of the eye ensure that the focus of the near reading vision is maintained on the retina.

Mice appear to lack the accommodation^12^ probably due to the poorly-developed ciliary muscles, the rigid lens and low requirement of visual resolution for the nocturnal animals^13, 14^. Therefore, the refractive status of mice should be less variable at different visual distances, compared to human eyes. In fact, the refractive status measured on same mouse eyes is significantly variable at different occasions of the measurement^2^. This result may be related to variations in eye orientation, intraocular pressure and position of the various optical interfaces during each individual measurement besides the possibility of measurement inaccuracy for the small eyes. Mydriasis with muscarinic antagonists has been used routinely prior to retinal examination^15^ and sometimes prior to refractive measurements of the mouse eye^2, 16–18^. It has been shown that mice could have a myopic shift occasionally during the refractive measurement after mydriasis with topical tropicamide^2^. In another study, 1% tropicamide solution produces a slight but significant hyperopic shift by about 1 D in the mice^19^. Daily topical administration of atropine for at least 2 weeks reduces axial growth of the mouse eye during normal visual environments^20^, suggesting that this detectable change in axial length of the eye may be responsible to the hyperopic shift. However, little is known about the mechanisms involved in this change from these studies.

During the mydriasis induced by topical use of muscarinic antagonists, both the thickness and curvature of the lens decrease, the anterior chamber depth increases and the axial length decreases in humans^21–23^, resulting in a hyperopic shift of the eye. Changes in choroidal thickness (ChT) during the topical administration of muscarinic antagonists was also observed in previous studies of human trials^21, 24–26^ with some studies reporting a thickening choroid^21, 26^ and others demonstrating a thinning choroid^24, 25^. These results indicate that posterior segment of the eye undergoes dimensional changes after treatment with muscarinic antagonists, which may contribute to the refractive changes during the mydriasis.

Biometric changes of the eye during topical administration of muscarinic antagonists have not been fully investigated in humans and mice though these changes have been well studied during accommodation in human trials^8–11^. It is impossible to directly assess accommodation in small animals with the method used for human trials as we cannot confirm whether the animal is focusing at an expected visual target. Therefore, an understanding of biometric changes under pharmacological manipulation could help explore whether the mice have any potential accommodation. Pharmacological induction of accommodation with muscarinic agonists is technically difficult for the mouse model due to the inaccuracy in refractive measurement for the eyes with a constricted pupil. Oppositely, if a muscarinic antagonist induced a refractive change in the mouse eye, which was resulted from changes in the axial dimension of the eye rather than in the anterior components, it would indicate that mice have no cycloplegia. This will indirectly suggest that mice have no potential accommodation and therefore further confirm the previous hypothesis which was only based on the anatomical assessment^13, 14^.

In this study, we assessed biometric changes of the eye under topical administration of muscarinic antagonists in mice and human subjects. It was not a parallel comparison of individual parameters between mice and humans as some parameters measured in humans could not be measured accurately in mouse eyes. We first investigated the effect of muscarinic antagonists (cyclopentolate) on whole ocular biometry (refraction, axial components and radius of curvature in various optical interfaces) in the C57BL/6 wild type mice. The whole ocular biometry including ChT in human eyes was also assessed during accommodation and after topical administration of cyclopentolate. We then analyzed changes of ocular biometry responsible to refractive changes in each of the species.

## Results

### Changes in mouse ocular biometry after the cyclopentolate treatment

**Figure 1:**
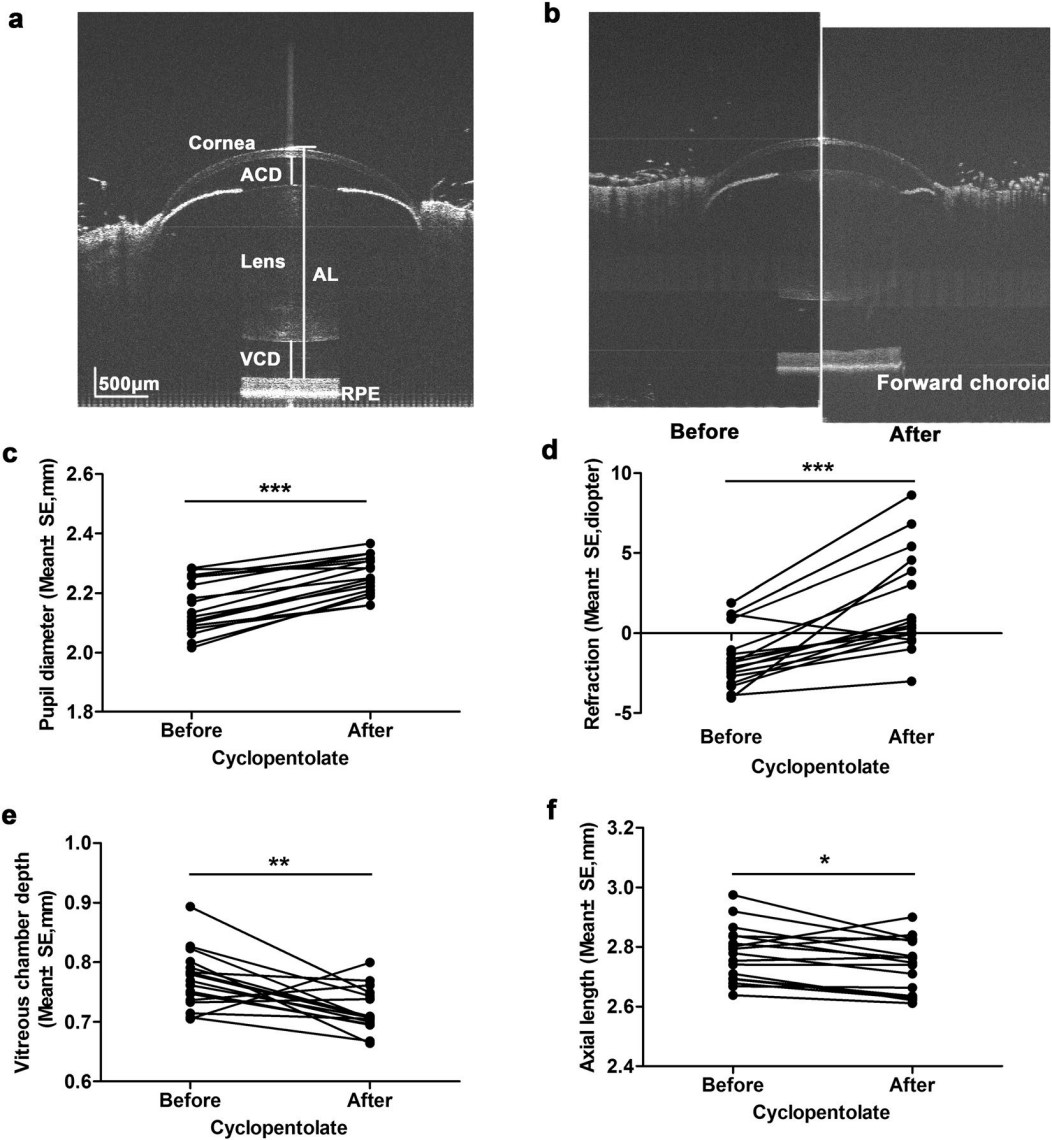
Changes in mouse ocular biometry after the cyclopentolate treatment. A scanned image of the mouse eye from the anterior cornea to the posterior retina was clearly obtained using spectral domain optical coherence tomography (**a**). Images of a mouse eye before and after the cyclopentolate treatment were trimmed and assembled together for comparison. The choroid was moved forward under the cyclopentolate compared to the pre-treatment, but the curvature of all optical interfaces and lens position did not change significantly (**b**). A significant larger pupil size (**c**), and a hyperopic shift (**d**) with a reduction in vitreous camber depth (**e**) and axial length (**f**) were induced in the cyclopentolate treatment. *P < 0.05, **P < 0.01 and ***P < 0.001, paired sample t-test.

No cornea dryness or lens opacification was observed during the biometric measurements in mouse eyes (Fig. 1a). The retinal pigment epithelium, as identified by the inner border of the choroid, moved forward in eyes treated with cyclopentolate compared to the pre-treatment, however the chorio-scleral interface was hardly detected (Fig. 1b). Pupil diameter in the cyclopentolate-treated eyes increased by 0.10 mm (2.26 ± 0.02 mm in cyclopentolate *vs* 2.16 ± 0.02 mm in pre-treatment: *P* < 0.001, paired sample t-test, Table 1, Fig. 1c). The eyes treated with cyclopentolate developed approximately 3.30 D hyperopia (1.76 ± 0.75 D in cyclopentolate *vs* −1.56 ± 0.44 D in pre-treatment: *P* < 0.001, Fig. 1d). The vitreous chamber depth in the cyclopentolate-treated eyes was reduced by 0.052 mm (0.719 ± 0.009 mm in cyclopentolate *vs* 0.771 ± 0.012 mm in pre-treatment: *P* = 0.003, Fig. 1e) and 0.041 mm for axial length (2.735 ± 0.022 mm in cyclopentolate *vs* 2.776 ± 0.023 mm in pre-treatment: *P* = 0.013, Fig. 1f). However, other axial components (central corneal thickness, anterior chamber depth, lens thickness and retinal thickness) or curvature of all optical interfaces (corneal or lens radius of curvature) did not change significantly in these eyes when compared to the pre-treatment (*P* > 0.05, Table 1).

**Table 1:** Changes in mouse ocular biometry after the cyclopentolate treatment (Mean ± SE).

Measured Parameters	Pre-treatment	Cyclopentolate
Pupil diameter (mm)	2.16 ± 0.02	2.26 ± 0.02***
Refraction (D)	−1.56 ± 0.44	1.76 ± 0.75***
Central corneal thickness (mm)	0.110 ± 0.002	0.107 ± 0.002
Anterior corneal radius of curvature (mm)	1.304 ± 0.032	1.336 ± 0.035
Posterior corneal radius of curvature (mm)	1.187 ± 0.058	1.236 ± 0.028
Anterior chamber depth (mm)	0.356 ± 0.006	0.363 ± 0.007
Lens thickness (mm)	1.541 ± 0.013	1.543 ± 0.013
Anterior lens radius of curvature (mm)	1.071 ± 0.081	1.112 ± 0.035
Posterior lens radius of curvature (mm)	0.904 ± 0.043	1.024 ± 0.085
Vitreous chamber depth (mm)	0.771 ± 0.012	0.719 ± 0.009**
Axial length (mm)	2.776 ± 0.023	2.735 ± 0.022*
Retinal thickness (mm)	0.226 ± 0.006	0.229 ± 0.004

^*^ *P* < 0.05, **P < 0.01 and ***P < 0.001 compared to pre-treatment, paired sample t-test.

### Changes in human ocular biometry under different treatments

**Figure 2:**
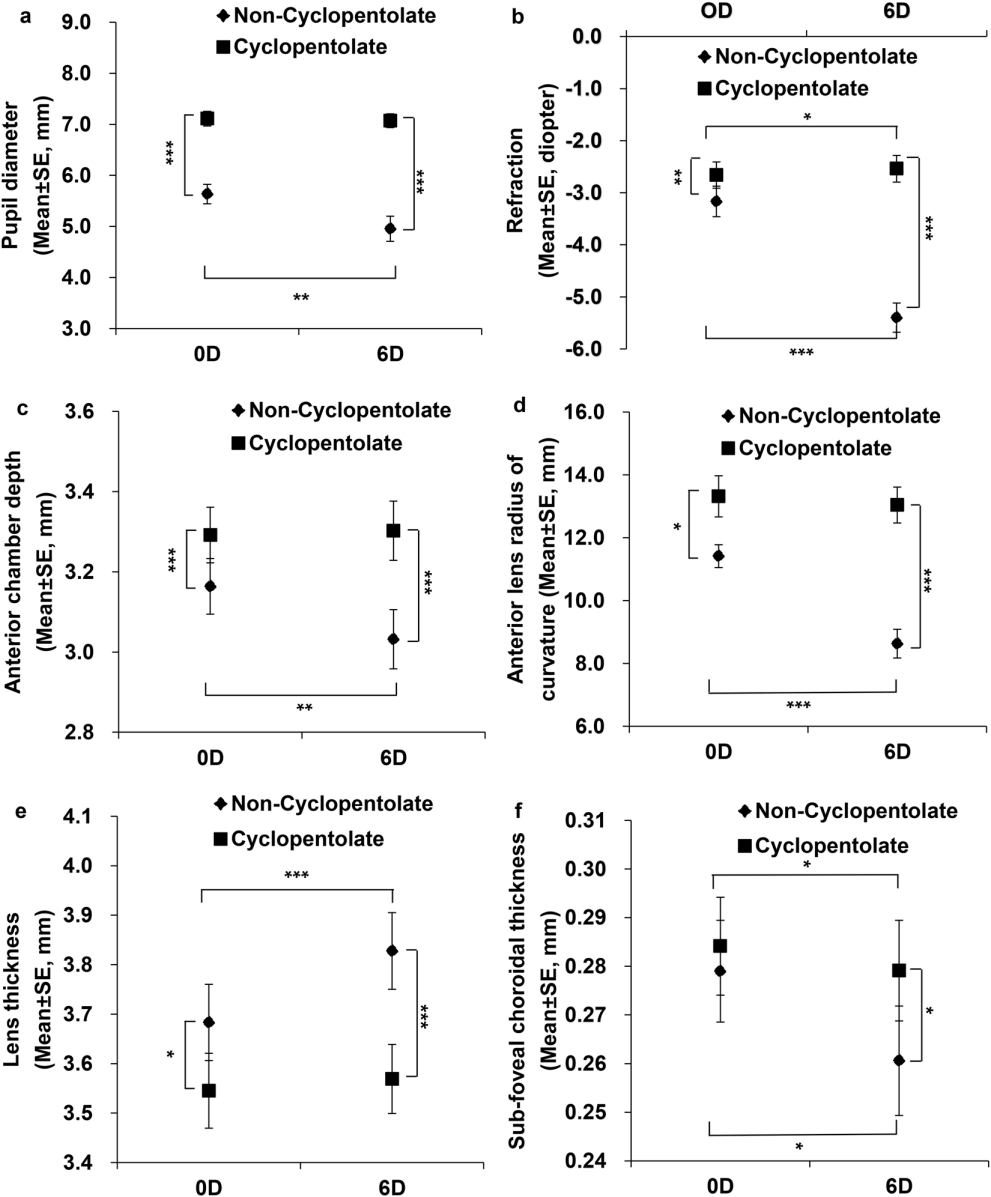
Changes in human ocular biometry under different treatments. Biometric measurements in Non-Cyc-0D, Non-Cyc-6D, Cyc-0D, and Cyc-6D groups during accommodative stimuli and after the cyclopentolate treatment. (**a**) Pupil diameter; (**b**) Refraction; (**c**) Anterior chamber depth; (**d**) anterior lens radius of curvature; (**e**) Lens thickness; (**f**) Sub-foveal choroidal thickness. *P < 0.05, **P < 0.001, two-way repeated ANOVA.

Two-way ANOVA showed that there was an interaction between accommodative stimuli and topical administration of cyclopentolate in determination of pupil diameter, refraction, accommodative response, anterior chamber depth, anterior lens radius of curvature, lens thickness, and ChT (*P* < 0.05, interaction, two-way repeated ANOVA, Table 2). Prior to the cyclopentolate treatment, the pupil diameter induced by the 6D accommodative stimulus was much smaller than by the 0D (*F*_*1,10*_ = 15.650, *P* = 0.003, post hoc simple effect analysis, Fig. 2a). The 6D accommodative stimulus induced a myopic shift of −5.40 ± 0.28 D with an accommodative response of -3.17 ± 0.26 D, while the Non-Cyc-0D induced a myopic shift of −3.17 ± 0.29 D and an accommodative response of −0.94 ± 0.12 D (*F*_*1,10*_ = 64.413*, P* < 0.001 for refraction; *F*_*1,10*_ = 64.379*, P* < 0.001 for accommodative response between these 2 groups, Fig. 2b). In parallel to the refractive changes, the anterior chamber depth was 3.032 ± 0.074 mm in Non-Cyc-6D and 3.164 ± 0.069 mm in Non-Cyc-0D (*F*_1,10_ = 21.454, *P* = 0.001, Fig. 2c). The anterior lens radius of curvature decreased significantly from 11.416 ± 0.364 mm in Non-Cyc-0D to 8.631 ± 0.455 mm in Non-Cyc-6D (*F*_1,10_ = 30.563, *P* < 0.001, Fig. 2d). The lens thickness increased significantly from 3.683 ± 0.077 mm in Non-Cyc-0D to 3.828 ± 0.077 mm in Non-Cyc-6D (*F*_1,10_ = 28.201, *P* < 0.001, Fig. 2e). ChT in Non-Cyc-6D decreased significantly from 0.279 ± 0.010 mm in Non-Cyc-0D to 0.261 ± 0.011 mm in Non-Cyc-6D (*F*_1,10_ = 8.109, *P* = 0.017, Fig. 2f). After the cyclopentolate treatment, there was only a small but significant difference in refraction, accommodative response and ChT between Cyc-0D and Cyc-6D (*P* < 0.05, Table 2). The other parameters were not affected by accommodative stimuli after the cyclopentolate treatment (*P* > 0.05).

**Table 2:** Changes in human ocular biometry under different treatments (Mean ± SE).

Measured Parameters	Non–Cyc–0D	Non–Cyc–6D	Cyc–0D	Cyc–6D
Pupil diameter (mm)	5.63 ± 0.19	4.96 ± 0.25**	7.11 ± 0.14***	7.07 ± 0.13^###^
Refraction(D)	−3.17 ± 0.29	−5.40 ± 0.28***	−2.66 ± 0.25**	−2.54 ± 0.26^†###^
Accommodative response (D)	−0.94 ± 0.12	−3.17 ± 0.26***	−0.43 ± 0.17**	−0.31 ± 0.18^†###^
Central corneal thickness (mm)	0.539 ± 0.008	0.535 ± 0.009	0.535 ± 0.011	0.532 ± 0.006
Anterior corneal radius of curvature (mm)	7.642 ± 0.149	7.913 ± 0.156	8.131 ± 0.150**	8.116 ± 0.110
Posterior corneal radius of curvature (mm)	6.788 ± 0.278	6.799 ± 0.246	7.061 ± 0.247	6.810 ± 0.188
Anterior chamber depth (mm)	3.164 ± 0.069	3.032 ± 0.074**	3.292 ± 0.069***	3.302 ± 0.071^###^
Lens thickness (mm)	3.683 ± 0.077	3.828 ± 0.077***	3.545 ± 0.075*	3.569 ± 0.070^###^
Anterior lens radius of curvature (mm)	11.416 ± 0.364	8.631 ± 0.455***	13.318 ± 0.653*	13.043 ± 0.574^###^
Posterior lens radius of curvature (mm)	6.072 ± 0.396	4.772 ± 0.653	6.593 ± 0.846	6.314 ± 0.576^#^
Vitreous chamber depth (mm)	18.549 ± 0.217	18.356 ± 0.265	18.314 ± 0.258	18.348 ± 0.258
Axial length (mm)	25.935 ± 0.188	25.780 ± 0.209	25.711 ± 0.249	25.751 ± 0.246
Retinal thickness(mm)	0.203 ± 0.003	0.208 ± 0.004	0.206 ± 0.004	0.203 ± 0.003
Choroidal thickness (mm)	0.279 ± 0.010	0.261 ± 0.011*	0.284 ± 0.010	0.279 ± 0.010^†#^

^*^ *P* < 0.05, **P < 0.01 and ***P < 0.001 compared to Non–Cyc–0D; ^†^ *P* < 0.05 compared to Cyc–0D; ^#^ *P* < 0.05 and ^###^ *P* < 0.001 compared to Non–Cyc–6D, post hoc simple effect analysis, two-way repeated ANOVA.

Cyclopentolate treatment in both Cyc-0D or Cyc-6D groups significantly decreased refraction, accommodative response and lens thickness and increased the pupil diameter, anterior chamber depth and anterior lens radius of curvature as compared to their control group (Cyc-0D *vs* Non-Cyc-0D, Cyc-6D *vs* Non-Cyc-6D, *P* < 0.05, two-way repeated ANOVA, post hoc simple effect analysis, Table 2). In addition, the anterior corneal radius of curvature in Cyc-0D was larger than in the Non-Cyc-0D (*F*_1,10_ = 11.302, *P* = 0.007) after the cyclopentolate treatment. The ChT and posterior lens radius of curvature in the Cyc-6D was greater than in the Non-Cyc-6D group (*F*_*1,10*_ = 7.373, *P* = 0.022 for ChT; *F*_1,10_ = 6.130, *P* = 0.033 for posterior lens radius of curvature).

Central corneal thickness, vitreous chamber depth, axial length, or retinal thickness was not affected by either the accommodative stimuli or cyclopentolate treatment during the measurements (*P* > 0.05, Table 2).

## Discussion

This study demonstrated that refraction of the mouse eyes shifted towards hyperopia after topical administration of muscarinic antagonists, with a reduction in both the vitreous chamber depth and axial length. However, curvature of all optical interfaces, lens position, or retinal thickness did not change significantly during the hyperopic shift. In human subjects, prior to the cyclopentolate treatment, a 6D accommodative stimulus produced a myopic shift with a reduction in anterior chamber depth, ChT and anterior lens radius of curvature and an increase in lens thickness. In contrast, the cyclopentolate treatment produced a hyperopic shift with an increased anterior chamber depth and anterior lens radius of curvature and a reduced lens thickness. Neither the vitreous chamber depth nor axial length changed significantly in human subjects after the treatment. Therefore, this hyperopic shift in the mice treated with cyclopentolate was not caused by cycloplegia of the eye as found in human subjects, but probably due to the forward movement of the choroid. This hypothesis is consistent with a forward movement of the retinal pigment epithelium in the mouse model and a thickening choroid in human eyes undergoing the same drug treatment for 6D accommodative stimulus in this study.

The pupil diameter is an important index in assessment of the accommodative condition in humans. In this study, the pupil diameter induced by the 6D accommodative stimulus was significantly smaller than by the 0D without mydriasis, indicating the accommodative response had occurred after this stimulus was initiated. After the cyclopentolate treatment, a hyperopic shift was detected with a maximized cycloplegia (complete pupil dilatation) in both the 0D and 6D stimulus groups, indicating that the accommodative power was significantly inhibited by the cyclopentolate.

Muscarinic antagonists induced a significant hyperopic shift with a decrease in vitreous chamber depth and axial length in the mice. Such a reduction in both the vitreous chamber depth and axial length mainly resulted from the forward movement of the retina as a single topical administration of muscarinic antagonists was unlikely to cause a detectable change in the sclera. The non-involvement of the anterior segments of the mouse eye in the refractive change could be associated with the poorly-developed ciliary body and a less flexible lens compared to humans and other mammals^13, 14^. Furthermore, the forward movement of choroidal inner border was detected in the mouse model, suggesting that the hyperopic shift in the mouse model was associated with a thickening choroid as detected in the human subjects. Rapid refractive changes have been found to be associated with transient changes of ChT in form deprivation and defocus-induced refractive errors in chicks^27^ and guinea pigs^28^. This suggests that a rapid refractive change is more related to transient changes in the position of optical interfaces inside the eye than anatomical changes of the eye. Further investigations with an increased penetration of OCT which can measure the entire ChT in C57BL/6 wild type mice^29, 30^ may help confirm the choroidal factor in drug-manipulated refractive changes.

Similar to previous studies in humans^3–5^, we found that accommodation was accompanied with a reduced anterior chamber depth and lens radius of curvature and an increased lens thickness. In this study, the accommodative response in the human eyes was −3.17 ± 0.26 D for 6D accommodative stimulus. This significant accommodation should be taken into account during routine assessment (no cyclopedia) of refractive status among young people. The accommodative response in the Non-Cyc-0D was about 0.50 D greater than in the Cyc-0D with a reduced anterior chamber depth and an increased lens thickness, indicating that there was a minimal tonic accommodation in the Non-Cyc-0D eye^31^. Interestingly, the sub-foveal ChT in Non-Cyc-6D was reduced compared to Non-Cyc-0D or Cyc-6D while the ChT in Non-Cyc-0D was not different from the Cyc-0D, indicating that a greater accommodative response was associated with a thinner ChT while tonic accommodation had no effect on ChT. Inversely, cyclopentolate increased ChT in human, which was similar to the effect of another muscarinic antagonist, homoatropine^21^. The ChT changes in human subjects under accommodative stimulus or cyclopentolate treatment were in a small but significant magnitude, suggesting the importance of ChT in the regulation of refractive development.

Recent studies have demonstrated thinning of the choroid during accommodation in humans^32–35^. The exact mechanism involved in this process is unclear and cannot be explained by the Helmholtz’s theory. It is hypothesized that the choroidal thinning during accommodation is likely due to a forward movement of the ora serrata resulting from an accommodation-induced ciliary muscle contraction^36, 37^. It has been reported that application of muscarinic antagonist leads to changes in uveal structures, including iris thickness and morphology of the iridociliary region^23^. The choroid, as a part of the uveal structure, its thickness can be changed by the relaxation of ciliary muscles induced by the instillation of muscarinic antagonist^38^. However, the unchanged anterior segment parameters of the mouse eye suggest that there is no ciliary relaxation during the mydriasis^13, 14^. Therefore, the positional change in mouse choroid after the mydriasis was probably due to a direct action on muscarinic receptors by the cyclopentolate at the posterior segments of the eye. This hypothesis is consistent with the fact that a long-term administration of atropine could inhibit form deprivation myopia by a non-cycloplegia mechanism in chicks as the ciliary muscle receptors are nicotinic in this species^39^. Muscarinic antagonists reduce the tension of collagenous and elastic connective tissues that spread over the choroidal stroma, thus limit the fluid transmission from the choroidal vessels to the surrounding tissues, leading to choroidal vasodilation^40^. Additionally, muscarinic receptors have been identified in a variety of vascular endothelium which act to regulate the vascular tone through relaxing factor such as nitric oxide^41, 42^.

The choroid could act as a temporary mechanism to regulate the axial length in animals^43, 44^. In order to compensate for retinal defocus, the choroid rapidly becomes thinner while the image is behind retina (hyperopic defocus) and thicker while the image is in front of the retina (myopic defocus)^43, 44^. Unlike small animals, transient changes in ChT may not have a significant impact on refraction in humans due to the much greater eye size. Additionally, the choroid, as a middle layer of the signal transmission between retina and sclera, could block biological signals originating from the retina^45, 46^ or secrete some new biological signals to affect the scleral and axial growth^47^. These actions from the choroid may present as changes in choroidal thickness. Previous studies in chicks or tree shrews have reported changes of gene expression in choroid during the development of myopia^48–50^, suggesting the choroid plays an active role in new molecular signals that alter scleral and axial growth of the eye. Further research is required to better understand the implications of accommodation-induced choroidal thinning in the development of human myopia.

In conclusion, we have demonstrated that mice do not have a traditionally-defined accommodation and the hyperopic shift induced by muscaric antagonists is not related to changes in anterior segment of the eye. It appears that this refractive change is due to a shortening vitreous chamber depth resulted from a forward thickening of the choroid. This may explain why refractions measured in mice are always more variable than in human subjects as choroidal thickness fluctuates more readily than other ocular components due to its vascular nature. It is important to know whether changes in choroidal thickness also play a significant role in long-term development of human myopia.

## Materials and Methods

### Ethics

The animal study was approved by the Animal Care and Ethics Committee at Wenzhou Medical University (Wenzhou, China). The experiments were conducted according to the ARVO Statement for the Use of Animals in Ophthalmic and Vision Research. Seventeen C57BL/6 wild type mice (age: 4–6 weeks equivalent to teenage in humans) were obtained from the animal breeding unit at Wenzhou Medical University. All animals were raised in standard mouse cages with a 12-hour light/dark cycle. The light provided by incandescent bulbs produced an ambient luminance of about 500 lux on the cage floor.

This study enrolled eleven human subjects aged from 23 to 33 years (25.36 ± 0.78 years) according to the tenets of the Declaration of Helsinki after its approval from the Ethics Committee of Wenzhou Medical University. All participants signed the informed consent prior to study participation. Human subjects with a degree of myopia were randomly selected in order to assess the power of accommodation in proportionate to the refractive errors measured. The mean spherical equivalent of refraction in all the subjects was −2.23 ± 0.33 D (range from −0.50 D to −3.75 D). The subjects had no systemic diseases, ocular diseases, laser ocular treatment or eye surgery.

### Mouse study design

Refraction, axial components and the radius of curvature in various optical interfaces of the mouse eye were measured prior to any treatment (baseline) and 45 minutes after topical administration of 1% cyclopentolate hydrochloride (Alcon, Belgium) with 3 interrupted drops at a 5-minute interval to achieve a complete pupil dilatation (pupil diameter: ~ 2.2 to 2.4 mm). Only the right eye of each animal was used for measurements.

Refraction of the mice was measured using an eccentric infrared photorefractor in a dimmed room (illuminance <2 lux) as described in details previously^2, 51, 52^. Briefly, the mouse was placed on a small platform in front of the photorefractor and gently positioned by grabbing its tail until the first Purkinje image was clearly shown in the center of the pupil (an indication of on-axis measurement). The data were then collected using software designed by Schaeffel^2^. The refraction was measured at least three times for each eye with the mean value recorded as the final result.

Biometric measurements were performed with a custom-made spectral domain OCT (Table 3) in mice and these included central corneal thickness, anterior chamber depth (distance between corneal endothelium and anterior lens surface), lens thickness, vitreous chamber depth (distance between posterior lens surface and internal limiting membrane of retina), retinal thickness, axial length (distance between anterior corneal surface and internal limiting membrane of retina), anterior and posterior corneal radius of curvature and anterior and posterior lens radius of curvature^51, 53^.

**Table 3:** Configuration of optical coherence tomography (OCT).

	Spectral domain	Ultra-long scan depth	Ultra-high resolution
Measurements	Mouse biometric components	Human axial components	Human retinal and choroidal thickness
Axial Resolution	6 μm	7.7 μm	3 μm
Scan Speed	24,000 Scans/s	17,500 Scans/s	70,000 Scans/s
Center Wavelength	840 nm	840 nm	850 nm
Band Width	50 nm	50 nm	100 nm
Scan depth in air	7.2 mm	37.71 mm	2.0 mm
Image Size of Each B-scan (pixels)	2048*1024	2048*12,288	2048 *1024

As described previously^52, 54, 55^, the spectral domain OCT system was based on the fiber-optics Michelson interferometer configuration (Table 3) with an axial resolution of ~ 6.0 μm. The scan depth of this OCT system was 7.2 mm in air and sufficient for scanning axial components of the mouse eye (~ 3mm axial length). Briefly, the mouse was gently restrained by grabbing its neck and placed on a platform in front of the OCT. The operator adjusted the position of the mouse eye until both the x- and y-cross scans appeared an indication of on-axis measurement. The OCT measurement usually lasted 3 minutes while the mouse was acclimated to this restrain during the measurement (no signs of restless, vocalization or body shaking). The raw OCT data (Fig. 1a) were exported and analyzed using a custom-designed software to obtain axial parameters and radius of curvature of various ocular components. A ray-tracing algorithm was used to correct image distortions due to refraction of the OCT beam at the successive boundaries of various ocular components.

### Human study design

Refraction, axial components including ChT and the radius of curvature in various optical interfaces of the left eyes were measured for all subjects during each of the following successive stages (1) Non–Cyc–0D: No drug treatment with the subject viewing a 0D stimulus, (2) Non-Cyc-6D: No drug treatment with the subject viewing a 6D stimulus, (3) Cyc-0D:the subject viewed a 0D stimulus after treated with the cyclopentolate, and (4) Cyc-6D: the subject tried to viewed a 6D stimulus after treated with the cyclopentolate. The accommodative stimulus was provided by a Badal system during the measurements. A white letter “E” on a black background was served as the fixation target and accommodative stimulus. As accommodation occurs in a binocular synchronization, the accommodative stimulus was induced in the contralateral (right) eye during ChT measurement on the left eye. For all other ocular parameters, the left eye was used for accommodative stimulation and measurements while the right eye was covered by an eye pad. The subjects were instructed to focus on the 0D or 6D accommodative target and maintain the target as sharp as possible. The muscarinic antagonist was administrated topically in the left eye with 3 interrupted drops of 1% cyclopentolate at a 5-minute interval to achieve a complete pupil dilatation (pupil diameters: ~ 7 to 8 mm).

Refraction was measured dynamically from the left eye under uncorrected condition using the Grand Seiko WAM–5500 auto-refractor (Grand Seiko Co. Ltd., Hiroshima, Japan) in HI-SPEED mode^56^. The accommodative response level was calculated by subtracting the spherical equivalent of refraction component of each subject from the data of the auto-refractor^57^. Three repeated refractive measurements were taken with the mean used for analysis.

Axial components including central corneal thickness, anterior chamber depth, lens thickness, vitreous chamber depth, axial length and the radius of curvature in various optical interfaces of the left eyes were measured using an ultra–long scan depth OCT, with a scan depth of 37.71 mm (Table 3)^58, 59^. In order to ensure that the measurement was conducted on the fixation/visual axis, the OCT measuring beam was congruent to the target. Measurements were repeated twice for each parameter by the same researcher. A custom-made software was used to reconstruct full eye images, and this software has been validated previously^58, 59^.

Both the retina and choroid in human subjects were imaged using an ultra-high resolution OCT, with an axial resolution of approximately 3 μm (Table 3). The OCT was adapted onto a slit-lamp system with the installation of an ocular lens (60 D; Volk Optical, Mentor, OH, USA) on the sample arm to detect the posterior segment of the eye. Two repeated measurements were taken by the same researcher for each parameter. The OCT images were exported from the instrument and analyzed using a custom-made software after data collection. The internal limiting membrane and retinal pigment epithelium initially was segmented using an automated method based on graph theory. An experienced observer then manually segmented the chorio-scleral interface using a method that has been previously described^60^. The software automatically applied a smooth function (spline fit) to define the boundary for these points.

### Statistics

A paired sample t-test was used to assess the change in mouse biometric components after treatment with cyclopentolate while two-way repeated ANOVA was used to analyze the change in human ocular biometry under different treatments. All data in this study were presented as Mean ± SE. Statistical significance was defined as *P < 0.05, **P < 0.01 and ***P < 0.001 (SPSS Version 16.0).

### Data Availability

The datasets generated and analysed during the current study are available from the corresponding author on reasonable request.
